# A Four-Layer Numerical Model for Transdermal Drug Delivery: Parameter Optimization and Experimental Validation Using a Franz Diffusion Cell

**DOI:** 10.3390/pharmaceutics17101333

**Published:** 2025-10-14

**Authors:** Fjola Jonsdottir, O. I. Finsen, B. S. Snorradottir, S. Sigurdsson

**Affiliations:** 1 Faculty of Industrial Engineering, Mechanical Engineering and Computer Science, University of Iceland, 107 Reykjavik, Iceland; oif1@hi.is (O.I.F.); sven@hi.is (S.S.); 2 Faculty of Pharmaceutical Sciences, University of Iceland, 107 Reykjavik, Iceland; bss@hi.is

**Keywords:** diffusion, mass transfer, mathematical modelling, partition, stratum corneum

## Abstract

**Background/Objectives:** A mechanistic understanding of transdermal drug delivery relies on accurately capturing the layered structure and barrier function of the skin. This study presents a four-layer numerical model that explicitly includes the donor compartment, stratum corneum (SC), viable skin (RS), and receptor compartment. **Methods:** The model is based on Fickian diffusion and incorporates interfacial partitioning and mass transfer resistance. It is implemented using the finite element method in MATLAB and calibrated through nonlinear least-squares optimization against experimental data from Franz diffusion cell studies using porcine skin. Validation was performed using receptor concentration profiles over time and final drug content in the SC and RS layers, assessed via tape stripping and residual skin analysis. **Results:** The model provided excellent agreement with experimental data. For diclofenac, the optimized partition coefficient at the SC–RS interface was close to unity, indicating minimal interfacial discontinuity and that a simplified three-layer model may be sufficient for this compound. **Conclusions:** The proposed four-layer framework provides a physiologically informed and generalizable platform for simulating transdermal drug delivery. It enables spatial resolution, mechanistic interpretation, and flexible adaptation to other drugs and formulations, particularly those with significant interfacial effects or limited lipophilicity.

## 1. Introduction

Transdermal drug delivery (TDD) systems are increasingly recognized for their potential to provide controlled and sustained therapeutic effects [1]. While experimental studies are indispensable for the development and evaluation of these systems, numerical modeling serves as a complementary tool that can enhance understanding and expedite the design process [2,3]. By simulating drug transport across skin layers, models can predict concentration profiles and systemic absorption, thereby informing formulation strategies and reducing reliance on extensive in vitro or in vivo experiments [4,5].

A critical aspect of TDD modeling is the accurate determination of permeation parameters, such as diffusion coefficients and partition coefficients between skin layers [6,7]. These parameters are often challenging to measure directly, particularly when experimental data are limited to the cumulative amount of drug permeated into the receptor compartment [8,9]. The absence of detailed concentration data within individual skin layers can lead to uncertainties in parameter estimation and, consequently, in model predictions.

The field of mathematical modeling in TDD predominantly relies on the application of Fick’s second law of diffusion, a second-order partial differential equation that describes how drug concentration evolves over time and space [10,11,12,13,14]. While some models may treat the skin as a single homogeneous layer, simplifying computations, this approach often lacks the spatial resolution needed to accurately capture drug dynamics across distinct skin layers [15,16]. In contrast, multi-layer models better reflect the complex structure of skin and its transport barriers, especially the barrier function of the stratum corneum (SC), which is widely acknowledged as the primary obstacle to dermal absorption [1,17,18,19]. Furthermore, homogeneous models cannot account for partitioning at the interface between the SC and the viable epidermis, a phenomenon that is well documented in experimental and theoretical studies [20,21,22].

Recent studies continue to expand the capabilities of transdermal diffusion modeling, particularly in the context of layered skin structures and experimental validation. Multilayer models have been applied in conjunction with microfluidic systems to simulate drug transport under both healthy and pathological conditions [23]. Multilayer diffusion models have also been used alongside advanced imaging techniques to validate drug penetration and accumulation within distinct layers [24]. Additionally, analytical and numerical studies of multi-layered diffusion systems continue to support the use of compartmental approaches for simulating transdermal delivery [25]. These recent developments further support the need for mechanistically detailed models that can accommodate structural heterogeneity and be informed by experimental data.

A range of computational strategies have been applied to implement these models, including finite element, finite difference, and finite volume methods. Finite element analysis, in particular, offers high numerical accuracy and geometric flexibility and has demonstrated strong agreement with in vivo pharmacokinetics when applied to multilayer skin models [26]. However, model accuracy often depends more on the availability of suitable validation data than on the mathematical formalism itself. Indeed, many published models significantly underestimate in vivo drug flux by up to 1–2 orders of magnitude due to uncertainties in permeation parameters and oversimplified assumptions about interfacial behavior [27].

Beyond diffusion-based framework, physiologically based pharmacokinetic (PBPK) models offer an additional layer of sophistication by integrating systemic physiological parameters with local transport dynamics [28,29]. These models are particularly useful in predicting whole-body pharmacokinetics following transdermal administration. However, spatially resolved diffusion models remain essential for characterizing local drug transport and for generating accurate boundary conditions to inform PBPK models.

The in vitro Franz diffusion cell system is a standard method according to OECD [30] for assessing in vitro transdermal delivery. It is widely used [31,32,33] to evaluate skin permeability and drug–formulation interactions. Beyond permeation studies, it supports formulation optimization, toxicity assessment, and quality-control testing.

In this study, we present a four-layer numerical model that explicitly represents the donor compartment (DC), the stratum corneum (SC), the remaining skin layers (RS), and the receptor compartment (RC). The model is based on Fickian diffusion and incorporates both partitioning and interfacial resistance at each layer interface [2,34,35]. It is numerically solved using the finite element method, a robust technique for handling discontinuous boundary conditions and heterogeneous material properties [12,34,36]. The model is calibrated and validated using experimental data obtained from Franz diffusion cell studies on porcine skin, including tape stripping to measure the quantity of drug in the SC and extraction and weighing to determine the amount retained in deeper skin layers [9]. This layered dataset allows us to examine how the inclusion of detailed spatial concentration data impacts the estimation of permeation parameters.

To extract these parameters, we apply a nonlinear least-squares optimization algorithm, which minimizes the difference between simulated and measured drug concentrations in the receptor and skin compartments. The optimization targets key parameters such as diffusion coefficients, partition coefficients, and interfacial mass transfer coefficients. We further assess the role of skin-layer discretization by comparing results from a three-layer model with those from the full four-layer formulation. By integrating numerical modeling, experimental validation, and data-driven parameter estimation, this work provides a comprehensive framework for analyzing and predicting drug transport in transdermal delivery systems.

## 2. Materials and Methods

### 2.1. Experimental Setup

The experimental data used to validate the numerical model in this study were obtained from a previously published work in which the authors investigated the transdermal diffusion of diclofenac using a Franz diffusion cell [9]. In that study, diclofenac sodium salt was applied to porcine skin mounted between the donor and receptor chambers. The receptor chamber was filled with phosphate-buffered saline (PBS, pH 7.4), maintained at 32 ± 1 °C, and magnetically stirred. Samples were collected at regular intervals over a total period of roughly 50 h, and the concentration of diclofenac in the receptor phase was quantified using high-performance liquid chromatography (HPLC).

**Figure 1:**
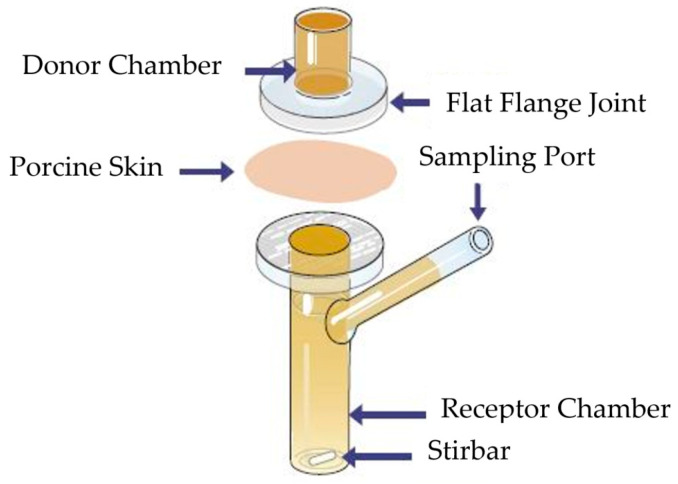
Schematic illustration of the Franz diffusion cell used in the experimental study. Adopted from [37].

Porcine ear skin, selected due to its physiological similarity to human skin, was dermatomed to a nominal thickness of approximately 1 mm. Franz diffusion cells used had an orifice diameter of 0.9 cm (exposure area 0.64 cm^2^), with a donor chamber volume of 1 mL and a receptor chamber volume of 12 mL. The receptor phase was degassed PBS. At time zero, 1 mL of a 5 mg/mL diclofenac sodium solution was applied to the donor compartment and sealed to prevent evaporation. Samples (200 µL) were withdrawn at predetermined time intervals and replaced with fresh PBS. A schematic illustration of a Franz diffusion cell is shown in Figure 1.

In addition to receptor sampling, tape stripping was performed at the end of the permeation study to quantify the amount of drug retained within the stratum corneum. After rinsing and drying the skin surface, adhesive tape strips were applied with uniform pressure and sequentially removed (70 times) to remove the SC layer. Tape strips were immersed in methanol, sonicated, centrifuged, and analyzed via HPLC to determine drug content.

The geometric and physical parameters of the experimental setup, including membrane thickness, receptor volume, and sampling frequency, were used to define the computational domain and boundary conditions in the numerical model. A summary of the experimental settings and HPLC methods can be found in Jónsdóttir et al. [9].

### 2.2. Mathematical Model

In a TDD system, the drug is loaded into the donor, which could be a patch, gel or cream for instance. It then undergoes passive diffusion from the donor and through the layers of the skin, ultimately entering the receptor compartment. The diffusion process is modeled with the well-known Fick’s second law. For a system with *i* layers in one-dimension, where the x-axis serves as the direction of diffusing molecules, Fick’s law within each layer can be expressed as:

(1) $$\frac{\partial C_{i}\left(x,t\right)}{\partial t}=\frac{\partial }{\partial x}\left(D_{i}\frac{\partial C_{i}(x,t)}{\partial x}\right)$$

where $C_{i}(x,t)$ is the concentration of dissolved drug within the *i*-th layer (mg/cm^3^) and $D_{i}$ is the coefficient of diffusion of drug in a dissolved state within the *i*-th layer (cm^2^/h).

**Figure 2:**
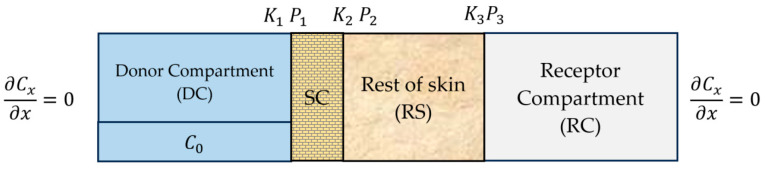
Schematic illustration of a four-layer transdermal diffusion model.

The computational domain used in this study consists of a four-layer system representing the key components of a typical transdermal drug delivery (TDD) setup. These layers are indexed by *i* = 1–4 for clarity in the mathematical formulation. Layer 1 corresponds to the donor compartment (DC), which acts as the drug reservoir. Layer 2 is the stratum corneum (SC), the outermost barrier of the skin. Layer 3 represents the rest of skin (RS), which includes the viable epidermis, dermis, and any residual subcutaneous fat that may have remained on the dermatomed porcine skin. Due to limited information on the exact anatomical separation and the small relative contribution of each, the drug amounts in these regions were summed and treated as a single diffusion domain labeled RS. Layer 4 is the receptor compartment (RC), representing the buffer solution in a Franz diffusion cell. A schematic illustration of the multilayer system is provided in Figure 2.

To solve the partial differential equation in (1) within each layer, both initial conditions and boundary conditions are required. The initial conditions specify the drug concentration at time t=0 in each layer:

(2) $$C_{1}(x,0)=C_{0},\;C_{2}\left(x,0\right)=0,C_{3}\left(x,0\right)=0,\;C_{4}\left(x,0\right)=0$$

The interlayer boundary conditions are defined to ensure continuity of flux across adjacent layers. However, a discontinuity in concentration may arise at the interface due to partitioning and interfacial resistance. In general, the boundary conditions at the interface between layers *i* and *i* + 1 are given as:

(3) $$-D_{i}{\left.\frac{\partial C_{i}\left(x,t\right)}{\partial x}\right|}_{x=x_{i}-}=-D_{i+1}{\left.\frac{\partial C_{i+1}\left(x,t\right)}{\partial x}\right|}_{x=x_{i}+}={K_{i}(C}_{i}(x_{i},t)-\frac{C_{i+1}(x_{i},t)}{P_{i}})$$

The notation $x_{i}^{-}$ and $x_{i}^{+}$ refers to the positions immediately to the left and right at the interface ${x=x}_{i}$, respectively. $K_{i}$ is the mass transfer coefficient between layers i and i+1 (cm/h), and $P_{i}$ is the partition coefficient between layers i+1 and i (-). In this work, the partition coefficient $P_{i}$ is defined as the ratio of drug concentration in the layer on the right to the concentration in the layer on the left at the interface ${x\;=x}_{i}$, that is,

(4) $$P_{i}=\frac{C_{i+1}(x_{i},t)}{C_{i}(x_{i},t)}$$

This convention ensures that $P_{i}>1$ indicates a preference of the drug to partition into the downstream layer, while $P_{i}<1$ indicates a preference for the upstream layer. Further note that $P_{1}$, $K_{1}$ represent the DC–SC interface, $P_{2}$, $K_{2}$ represent the SC–RS interface, and $P_{3}$, $K_{3}$ represent the RS–RC interface.

At the DC–SC and SC–RS interfaces, we assume $K_{1}$ and $K_{2}$ are large, leading to quasi-equilibrium conditions. As $K_{i}\to \infty ,$ the interface condition reduces to the partition condition:

(5) $$C_{i}\left(x_{i},t\right)-\frac{C_{i+1}\left(x_{i},t\right)}{P_{i}}=0$$

This assumption does not hold at the RS–RC interface, where a finite $K_{3}$ is used to model interfacial resistance. Finally, there is no flux from the donor to the external environment, nor from the receiver to the surrounding environment, as shown in Figure 2.

In summary, the four-layer model involves five unknown permeation parameters: three partition coefficients $P_{1}$, $P_{2}$, and $P_{3};$ one mass transfer coefficient $K_{3}$; and the diffusion coefficient of the SC${,\;D}_{SC}$. The diffusion coefficient for the rest of skin${,\;D}_{RS},$ is measured experimentally [9]. The donor and receptor compartments are assumed to be well-mixed, and their diffusion coefficients are therefore set to large values, representing a uniform drug distribution.

### 2.3. Numerical Implementation

To model drug diffusion across the four-layer transdermal system, Fick’s second law was solved numerically using the finite element method implemented in MATLAB R2024a. The problem was formulated in one spatial dimension, assuming homogeneous and isotropic material properties within each layer.

The spatial domain was discretized into linear 1D elements, and time integration was performed using an implicit backward Euler scheme, which provides numerical stability for the transient solution. Interfacial boundary conditions, incorporating both partitioning and finite mass transfer resistance, were applied at the internal interfaces between layers. These conditions, together with the initial and external boundary specifications described previously, ensure conservation of mass while allowing for discontinuities in concentration due to interfacial phenomena.

Simulations were run up to the final experimental sampling time. Drug concentration profiles were computed throughout the domain at each time step, and the cumulative drug mass in each layer was obtained via spatial integration. Mesh density and time step size were selected based on convergence testing to ensure accurate and stable numerical performance. Model predictions were then compared against experimental data for validation.

### 2.4. Parameter Estimation with Optimization

To estimate the permeation parameters that best match experimental observations, a nonlinear least-squares optimization approach was implemented in MATLAB’s lsqnonlin function. Key parameters such as diffusion coefficients, partition coefficients, and interfacial mass transfer coefficients govern the transport kinetics across the skin. Optimizing these parameters ensures accurate replication of experimental data and allows the model to be tailored for drug-specific behavior. The objective function minimized the sum of squared differences between simulated and experimentally measured drug concentrations in the receptor compartment and within the skin layers, specifically, the SC and the RS. This approach allowed for simultaneous fitting to multiple data sources, including receptor sampling over time and endpoint tape-stripping measurements.

The parameters subject to optimization included the diffusion coefficient in the SC ($D_{SC}$), partition coefficients across each interface ${(P}_{1}$, $P_{2}$, $P_{3}$) and the mass transfer coefficient at the RS-RC interface ($K_{3}$). Initial parameter guesses were based on prior studies or physiologically plausible values, and upper and lower bounds were applied to ensure numerical stability and realistic outputs. The optimization algorithm used a tolerance of 1 × 10^−5^ for both the step size (StepTolerance) and the residual norm (FunctionTolerance). These settings were chosen to ensure convergence without excessive computation time. In some cases, additional weights were applied to late-time receptor values to improve the fit in that region, especially when small deviations in later time points could significantly impact model validation.

## 3. Results and Discussion

### 3.1. Optimized Parameters and Model Fit

From the experiments, we obtained drug mass measurements in the receiver compartment at selected time intervals, as well as in the stratum corneum (SC), the rest of the skin (RS), and the donor at the final time point (51.5 h). These values are presented in Table 1 and represent the mean of all experimental replicates. These values serve as key validation points for our model. While minor variations exist between individual runs, due to differences in porcine skin samples, application consistency, and analytical sensitivity, the values reflect the typical distribution of drug mass across compartments. The model validation accounts for this variability by comparing simulated profiles to the overall experimental trends rather than exact individual measurements.

Simulations were carried out using the optimized permeation parameters obtained through nonlinear least-squares fitting. The optimization procedure minimized the sum of the squared residuals between the simulated results and experimental data, including drug concentrations in the RC over time and endpoint measurements from the SC, RS and DC. For the skin layers, residuals were calculated by comparing the median concentration within each layer, as estimated from the finite element model, with the corresponding experimental values. The use of the median concentration ensures unit consistency across compartments and reduces the influence of localized concentration peaks, thereby providing a more representative and robust measure for model fitting. A comparison of weighted vs. unweighted optimization showed only marginal differences in output, but the weighted version provided slightly better agreement with late-stage receptor data. Therefore, the weighted results are presented in the main text

Case 1: A logical starting point was to set the partition coefficient at the SC–RS interface ($P_{2}$) to unity, effectively collapsing the SC and RS into a single layer. This configureation reflects a three-layer model by assuming no concentration discontinuity at the SC–RS interface. Table 2 presents the resulting optimized parameters.

**Figure 3:**
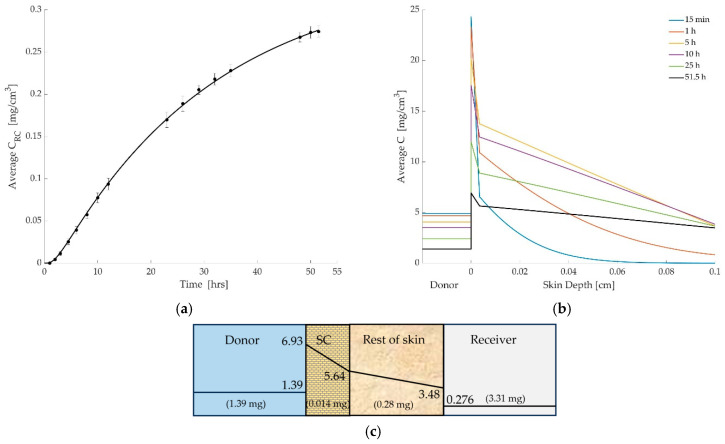
(**a**) Comparison between simulated and experimental drug concentrations in the receiver compartment over time. The solid line represents model predictions based on the optimized permeation parameters, while the circular markers with error bars denote experimental measurements (mean ± SD). (**b**) Simulated concentration profiles of diclofenac across the donor and skin layers at multiple time points (15 min to 51.5 h). The donor compartment is shown for reference but is not drawn to scale. The dashed vertical line indicates the thickness of the stratum corneum (SC), beyond which the rest of skin region begins. The figure illustrates the steep concentration drop across the SC, highlighting its role as a diffusion barrier. (**c**) The drug concentration (mg/cm^3^) within each layer and total drug mass (mg) per layer at time of 51.5 h.

The outcomes, using these parameters, are presented in Figure 3a–c below. The simulation results showed strong agreement with experimental data. Figure 3a compares the simulated and experimental cumulated mass of diclofenac in the receptor compartment over time. The model captures the initial lag time due to SC barrier function and the subsequent steady accumulation of drug in the receptor. Figure 3b shows the simulated spatial distribution of diclofenac across the skin layers at various time points. Figure 3c shows the mass retained in each compartment after time of 51.5 h.

Case 2: In the second scenario, all permeation parameters were optimized, including P_2_. This allowed the model to independently resolve the concentration discontinuity across the SC–RS interface. The final optimized values are provided in Table 3.

**Figure 4:**
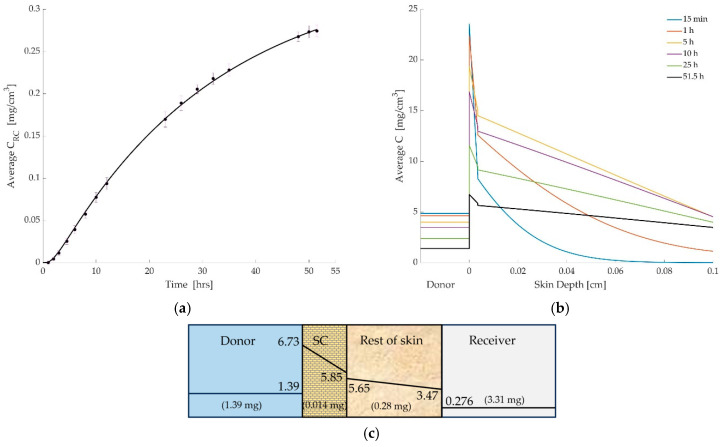
(**a**) Comparison between simulated and experimental drug concentrations in the receiver compartment over time. The solid line represents model predictions based on the optimized permeation parameters, while the circular markers with error bars denote experimental measurements (mean ± SD). (**b**) Simulated concentration profiles of diclofenac across the donor and skin layers at multiple time points (15 min to 51.5 h). The donor compartment is shown for reference but is not drawn to scale. The dashed vertical line indicates the thickness of the stratum corneum (SC), beyond which the rest of skin region begins. The figure illustrates the steep concentration drop across the SC, highlighting its role as a diffusion barrier. (**c**) The drug concentration (mg/cm^3^) within each layer and total drug mass (mg) per layer at time of 51.5 h.

The results again closely matched experimental data, as shown in Figure 4a. The four-layer formulation offered a modest improvement in capturing the early-time lag associated with SC resistance, and slightly adjusted the predicted concentrations within the skin layers. Figure 4b,c shows the time-resolved spatial profiles and cumulative drug mass per compartment at the endpoint.

The two cases yield similar overall trends, particularly in the cumulative drug uptake by the receptor compartment. However, the full four-layer model provides additional flexibility to characterize interface effects and capture early-time dynamics more accurately. Interestingly, the optimized P_2_ value was found to be close to unity. This suggests that the concentration of diclofenac at the SC–RS boundary is nearly continuous, and that the SC–RS interface does not impose a significant additional resistance to drug transport beyond what is already described by the diffusion coefficients within each layer.

This result carries two important implications. First, it validates the flexibility of the four-layer framework: although the model permits discontinuous conditions at the interface, the numerical solution adapts to the experimental data and reflects near-continuity where appropriate. Second, it suggests that for this particular drug formulation system, a three-layer model (in which the SC is treated as a thin boundary or lumped into a simplified skin layer) may produce comparable results, provided that such simplification is informed by a more general framework. Nevertheless, this outcome should not be interpreted as a limitation of the four-layer approach. Rather, it underscores its generality and adaptability. In systems involving drugs with different lipophilicity, molecular size, or formulation properties, significant partitioning at the SC–RS interface may emerge. The present result emphasizes that such partition behavior is drug-specific and must be assessed through model-based parameter estimation rather than assumed a priori.

### 3.2. Comparison with Three-Layer Models and Literature

In this work, we adopt a four-layer model that explicitly includes the stratum corneum (SC) as a distinct diffusive domain. This approach offers a more mechanistic representation of transdermal drug transport by isolating the SC, the principal barrier to permeation, and treating interfacial discontinuities using partition coefficients. This provides a clearer physical interpretation of transport behavior compared to traditional three-layer models, which often collapse the SC and viable skin (VS) into a single composite layer.

In three-layer models, any additional resistance at unresolved internal boundaries, such as between the SC and RS, is typically absorbed into empirical mass transfer coefficients at the outer interfaces (e.g., donor–skin or skin–receptor). These coefficients effectively combine effects from both true interfacial partitioning and possible resistance to mass transfer, but without explicitly resolving the underlying structure. As a result, such formulations may obscure the contribution of individual layers and interfaces, limiting physiological interpretability and transferability across drugs or systems.

Our results for diclofenac show that the optimized partition coefficient at the SC–RS interface (P_2_) was close to unity, indicating minimal discontinuity in concentration across this interface. This suggests that for this specific compound, the SC–RS interface contributes relatively little additional resistance beyond the internal diffusive properties of the layers. Thus, for certain drugs and formulations, a well-calibrated three-layer model may be sufficient. However, this outcome does not diminish the relevance or necessity of the four-layer approach. Rather, it highlights its adaptability, the model can confirm when simplifications are valid and allows for their justification through parameter estimation, rather than assumption. One of the strengths of the four-layer formulation lies in its exclusive use of partition coefficients to represent interface behavior. Partition coefficients are dimensionless, reflecting thermodynamic equilibrium across layers, and can be readily compared across drugs or formulations. In contrast, mass transfer coefficients have dimensional units and may reflect a mix of physical, geometrical, or experimental factors such as local microstructure or hydrodynamic conditions. When both partition and mass transfer coefficients are needed to describe a single interface, as is often the case in three-layer models, it becomes more difficult to assign precise physiological meaning to each parameter.

The literature supports this layered diffusion framework. Several studies have developed multi-layer models emphasizing the importance of representing the SC as a separate diffusive domain and capturing interfacial partitioning explicitly [38]. Similarly, other works highlight that for hydrophilic or charged drugs, the SC–VS interface can critically influence accumulation and penetration profiles [39]. More recent studies, such as [40], have demonstrated how inverse mechanistic modeling can be used to extract permeation parameters from experimental data, supporting the trend toward more physiologically grounded models of transdermal delivery. Finite element models based on layered skin geometry have also been shown to provide strong predictive performance, particularly when validated with experimental data [26]. However, even physiologically informed models frequently underestimate in vivo flux by up to two orders of magnitude, underscoring the need for careful parameter estimation and validation [27].

While our findings show that a four-layer model offers only modest improvements in predictive performance for diclofenac, the framework’s full potential emerges when applied to drugs with stronger interfacial partitioning or limited lipophilicity, where simpler models may fail to capture key transport mechanisms. Hydrophilic drugs such as caffeine and lidocaine tend to show more pronounced partitioning behavior at the SC–viable skin interface, often resulting in steeper concentration gradients due to their limited lipophilicity [41,42,43]. Additionally, the availability of layer-specific experimental data, such as tape stripping or residual skin content measurements, supports the four-layer model’s structure and enables direct model validation.

In summary, the four-layer model not only offers predictive accuracy, but also provides mechanistic clarity, making it a valuable tool for hypothesis testing, model validation, and extension to other drug formulation systems where interfacial phenomena play a more prominent role.

## 4. Conclusions

This study presents a four-layer finite element model for simulating transdermal drug delivery, explicitly representing of the donor compartment, stratum corneum (SC), viable skin, and receptor compartment. The model incorporates Fickian diffusion with interfacial partitioning and resistance, and estimates key permeation parameters through nonlinear optimization against experimental data, including Franz diffusion cell measurements and tape stripping.

For diclofenac, the optimized parameters showed strong agreement with measured concentration profiles. The estimated partition coefficient between the SC and viable skin was close to unity, suggesting limited interfacial resistance for this compound. While a simplified three-layer model may suffice in such cases, the four-layer framework provides a more mechanistic and adaptable approach that can account for drug-specific interfacial behavior when needed.

Importantly, this modeling approach enables spatial resolution, interface-specific parameterization, and validation against layer-specific data. These features are particularly relevant for hydrophilic or charged compounds, where interfacial effects are more pronounced. The framework thus offers a generalizable platform for hypothesis testing, formulation design, and regulatory evaluation of transdermal drug products.

Future perspectives: While this study focused on diclofenac as a model compound, future work will extend the four-layer framework to drugs with differing physicochemical properties, such as hydrophilic or ionized molecules, where interface partitioning may play a larger role. Incorporating experimental data from additional drugs and formulations will support broader validation and refinement of the model. The framework also lends itself to coupling with physiologically based pharmacokinetic (PBPK) models to simulate systemic absorption. Ultimately, this approach may support more accurate in silico screening and regulatory evaluation of transdermal delivery systems.

## Figures and Tables

**Table 1:** Experimental drug mass in each compartment after 51.5 h.

Donor (mg)	SC (mg)	Rest of Skin (mg)	Receiver (mg)
1.41	0.0140	0.280	3.29

**Table 2:** Optimized permeation parameters obtained by simulation for Case 1 ($P_{2}=1.00$).

Diffusion coefficient D_SC_	1.03 × 10^−4^ cm^2^/h
Diffusion coefficient D_RS_ (measured)	1.80 × 10^−3^ cm^2^/h
Partition coefficient P_1_	4.97
Partition coefficient P_2_	1.00
Partition coefficient P_3_	0.102
Mass transfer coefficient K_3_	0.054 cm/h

**Table 3:** Optimized permeation parameters obtained by simulation for Case 2.

Diffusion coefficient D_SC_	1.49 × 10^−4^ cm^2^/h
Diffusion coefficient D_RS_ (measured)	1.80 × 10^−3^ cm^2^/h
Partition coefficient P_1_	4.84
Partition coefficient P_2_	0.96
Partition coefficient P_3_	0.111
Mass transfer coefficient K_3_	0.043 cm/h

## Data Availability

The data presented in this study are available on request from the corresponding author.
